# Dietary Fiber as Prebiotics: A Mitigation Strategy for Metabolic Diseases

**DOI:** 10.3390/foods14152670

**Published:** 2025-07-29

**Authors:** Xinrui Gao, Sumei Hu, Ying Liu, S. A. Sanduni Samudika De Alwis, Ying Yu, Zhaofeng Li, Ziyuan Wang, Jie Liu

**Affiliations:** 1 Key Laboratory of Geriatric Nutrition and Health (Beijing Technology and Business University), Ministry of Education, Beijing 100048, China; 2 Shenzhen Key Laboratory of Metabolic Health, Center for Energy Metabolism and Reproduction, Shenzhen Institutes of Advanced Technology, Chinese Academy of Sciences, Shenzhen 518055, China; 3 School of Food Science and Technology, Jiangnan University, Wuxi 214122, China

**Keywords:** dietary fiber, prebiotics, intestinal disorders, metabolic diseases, molecular mechanisms

## Abstract

Dietary fiber (DF) is one type of carbohydrate that cannot be digested by the gastrointestinal tract. It is widely recognized as an essential ingredient for health due to its remarkable prebiotic properties. Studies have shown that DF is important in the management of metabolic diseases, such as obesity and diabetes, by regulating the balance of gut microbiota and slowing down the absorption of glucose. It is worth noting that patients with metabolic diseases might suffer from intestinal dysfunction (such as constipation), which is triggered by factors such as the disease itself or medication. This increases the complexity of chronic disease treatment. Although medications are the most common treatment for chronic disease, long-term use might increase the financial and psychological burden. DF as a prebiotic has received significant attention not only in the therapy for constipation but also as an adjunctive treatment in metabolic disease. This review focuses on the application of DF in modulating metabolic diseases with special attention on the effect of DF on intestinal dysfunction. Furthermore, the molecular mechanisms through which DF alleviates intestinal disorders are discussed, including modulating the secretion of gastrointestinal neurotransmitters and hormones, the expression of aquaporins, and the production of short-chain fatty acids.

## 1. Introduction

**Figure 1:**
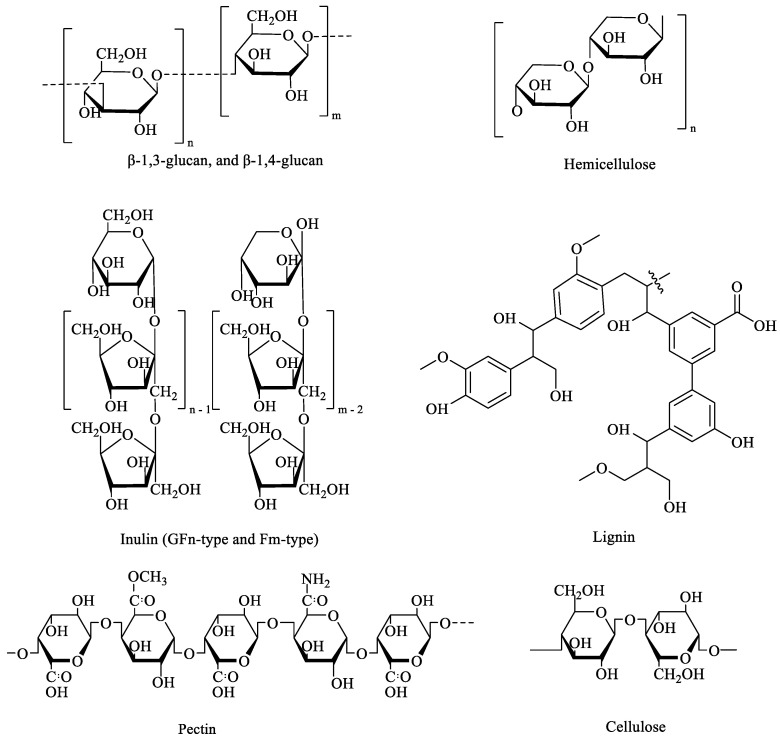
The structures of DF, including SDF (such as β-glucan, inulin, and pectin) and IDF (such as hemicellulose, cellulose, and lignin).

DF is defined as “the carbohydrates with 10 or more degrees of polymerization, which cannot be hydrolyzed by the enzymes of the human small intestine, but possess healthy benefits for the human body” [1]. The physicochemical properties of DF, such as solubility, viscosity, and thermal stability, as well as its biological activity, are significantly influenced by its molecular structure, including molecular weight, monosaccharide composition, functional groups, chain architecture, and conformation [2]. It is generally classified into insoluble dietary fiber (IDF) and soluble dietary fiber (SDF) based on the difference in solubility (Figure 1) [3,4]. IDF is one type of fiber that is insoluble in water and indigestible in the small intestine or non-fermentable in the colon. It generally includes cellulose, hemicellulose, lignin, and resistant starch (RS). IDFs have a hydrophobic structure consisting of hydrogen bonds between sugar chains [5]. They exert beneficial effects on the human body through physicochemical properties, such as good water-holding capacity, water swelling capacity, and adsorption capacity [6]. SDF is one type of water-soluble fiber that can be used by the gut microbiota in the colon. It includes oligosaccharides (β-glucan, fructans, and arabinose) and some indigestible polysaccharides (inulin, gum Arabic, gum, and pectin) [7]. The physiological functions of SDF are largely influenced by two key properties: viscosity and fermentability, of which viscosity is closely associated with molecular weight [8]. SDFs with high molecular weight (β-glucans and pectins) typically exhibit higher viscosity, which can delay gastric emptying and slow glucose absorption. In contrast, low-molecular-weight SDFs (inulin and short-chain fructooligosaccharides) are rapidly fermented by gut microbiota, serving as prebiotics that stimulate the growth of beneficial bacteria, such as *Lactobacillus* and *Bifidobacterium* [9]. These fermentation processes produce short-chain fatty acids (SCFAs), which have positive effects on maintaining intestinal health and alleviating metabolic diseases [10].

Notably, the molecular structures of DF vary significantly depending on their botanical source, which directly affects their physicochemical behavior and physiological functions. For example, cellulose from plant cell walls has a highly crystalline, linear structure with β(1→4) glycosidic bonds, making it highly resistant to enzymatic degradation [11]. Pectin from fruits, primarily composed of α-1,4-D-galacturonic acid residues, is more easily degraded and fermented [12]. RS in legumes exhibits dense crystalline granules and limited surface hydration, making it poorly accessible to enzymatic hydrolysis [13]. Alginate from seaweeds consist of linear β-(1→4) linkages between residues, which reduce their fermentability by gut microbiota [14]. Therefore, understanding the structure–function relationship of DF is crucial for performing the prebiotics function to prevention metabolic diseases.

Metabolic diseases pose a major challenge to global health systems, with prevalence rates steadily rising [15]. Studies have shown a significant association between higher DF intake and lower risk of metabolic diseases [16,17]. It has become an increasingly popular trend to reduce the incidence of metabolic diseases by dietary management, such as increasing DF intake. Complications arising from metabolic diseases should not be overlooked, as they are often accompanied by long-term treatment, such as intestinal dysfunction. Metabolic diseases and intestinal dysfunctions are bi-directionally regulated: metabolic abnormalities can affect intestinal function through neurological, immunological, and endocrine pathways, and imbalances in intestinal microecology and intestinal function may exacerbate metabolic disorders [18]. Multi-ethnic cohort studies have shown that approximately 70–75% of patients with diabetes report at least one gastrointestinal symptom [19]. Constipation is the most common intestinal disorder in diabetic patients, with a prevalence of 15.0–24.5% [19]. While most studies have focused on the effects of DF on metabolic diseases, there are currently no studies investigating its impact on metabolic diseases and intestinal dysfunction. Here, we summarized the application of DF in regulating metabolic diseases with a focus on how DF exerts a positive role in intestinal function from the perspective of molecular mechanisms.

## 2. The Health Benefits of DF in Alleviating Metabolic Diseases

Probiotics and prebiotics have been found to improve metabolic diseases through regulating gut bacteria metabolism [20]. DF, in addition to bacterial regulation, also provides synergistic interventions through physicochemical properties and inhibition of inflammatory responses [21]. For instance, consuming DF has been shown to help alleviate obesity, diabetes, and cardiovascular diseases (CVDs) [22,23]. Intestinal disorders, especially constipation, are the common complication of metabolic diseases. Here, we explored the effect of DF on metabolic diseases and their associated intestinal complications.

### 2.1. Prevention of Overweight and Obesity

Globally, over 39% of adults are overweight and 13% obese, significantly increasing risks of insulin resistance and constipation [24,25]. The role of DF in weight control stems not only from its physicochemical properties but is also closely related to its prebiotic properties [26]. Overweight and obese patients consuming SDF for 12 weeks not only experienced significant reductions in body weight and waist circumference but also increased the abundance of *Bifidobacteria* in the gut [27]. In another trial of 37 overweight or obese participants, after 8 weeks of RS supplementation, participants lost weight by an average of 2.8 kg and had a significant increase in *B. adolescentis* [28]. According to a trial involving 62 studies on the effects of viscous fiber intake on body weight and obesity, increased fiber intake enhanced the viscosity of gut contents, reduced nutrient kinetics and absorption, and delayed gastric emptying [29]. These studies suggested that the change in body weight might occur through the synergistic effect of DF and gut microbiota. Propionate is one of the metabolites of gut microbiota, which enhances feelings of satiety [30]. In a study carried out on overweight adults, participants who consumed arabinoxylan (AX; a purified fiber with medium viscosity and fermentability) for six weeks showed an increase in satiation along with an increased proportion of propionate [30]. In addition, a number of appetite-related gastrointestinal hormones are modulated by DF, which decreases the secretion of ghrelin and increases the release of cholecystokinin (CCK), glucagon-like peptide-1 (GLP-1), and peptide YY (PYY) [31]. For example, the PYY in serum is increased by consumption of β-glucan-rich bread or a DF-containing dinner [32]. In summary, DF may reduce body weight through slowing energy absorption, delaying gastric emptying, colonizing probiotics, producing gut bacteria metabolites, and modulating gastrointestinal hormones [29,33].

### 2.2. Prevention of Diabetes and Reduction of Blood Glucose Levels

The prevalence of diabetes is predicted to rise to 12.2% by 2045, which will increase the prevalence of digestive complications, including gastroparesis and intestinal disorders [33]. In a double-blind trial investigating the effects of pectin-containing compounds on postprandial glycemic response in healthy adults, the intervention group demonstrated significantly improved blood glucose control, which might be associated with an increase in GLP-1 levels [34]. In another clinical trial of 20 pre-diabetic patients who were offered an oral dietary supplement consisting of SDF and slow-digesting isomaltulose (LC-ONS), patients who consumed LC-ONS had significantly lower blood glucose levels and an increased area under the insulin curve, demonstrating that slow-digesting DF reduced the rate of postprandial blood glucose rise [35]. In addition to controlling the rise of blood glucose, DF improves serum metabolism in diabetic patients by affecting gut microbiota [36]. Providing a high-fiber diet to patients with diabetes resulted in increases in the abundance and diversity of intestinal bacterial communities and the levels of insulin and C-peptide, as well as significant reductions in serum glycosylated hemoglobin (HbA1c) and fasting blood glucose levels [36]. When providing diabetes patients with either normal food or a high-fiber diet, the high-fiber diet group showed greater abundance of gut microbiota and an increase in GLP-1, while a decrease in HbA1c [37]. Therefore, the effects of DF on diabetes may be through stimulating the secretion of digestive juices and absorption of water, leading to increased bloating and satiety, thus slowing down glucose absorption [38].

### 2.3. Prevention of CVD and Improvement of Dyslipidemia

CVD is one of the leading causes of death worldwide and might be treated by intervention of prebiotics [10,39]. As one type of SDF, diets supplemented with psyllium were effective in reducing low-density lipoprotein cholesterol (LDL-C) in adolescents [40]. In addition, studies showed that intake of psyllium for seven weeks reduced both small-dense LDL (sdLDL) and interleukin 6 (IL-6) levels, which is an independent predictor of CVD [41,42]. DF with high molecular weights has better viscosity due to the binding forces between the fiber molecules being sufficient to overcome external stresses. Comparison of the physicochemical and functional properties of several SDFs revealed that konjac glucomannan (KGM) had the best viscosity and exhibited good cholesterol absorption [43]. Dyslipidemia is a risk factor for cardiovascular disease, which might be prevented by altering the composition and function of the gut microbiota [44]. In a double-blind trial of 39 people, daily consumption of a mixture enriched with 7 kinds of DF for 8 weeks resulted in modulated gut microbiota, lowered total cholesterol and LDL cholesterol levels, and alleviated dyslipidemia [44]. Cholesterol serves as a precursor to bile acids (BAs). DF reduces blood cholesterol levels by promoting BA metabolism through adsorption of BAs via hydrophobic interactions, and generation of SCFAs that activate G-protein-coupled receptors (GPCRs) in intestinal epithelial cells to enhance BA excretion [45,46]. DF may prevent CVD through increasing intestinal luminal viscosity, improving cholesterol composition, decreasing indicators of inflammatory factors, altering the composition of gut microbiota, as well as promoting bile acid excretion [44,47].

### 2.4. Bowel-Function-Related Metabolic Diseases

Metabolic diseases impact gastrointestinal structure and function through multiple pathways, leading to symptoms such as constipation, diarrhea, gastroparesis, and inflammatory bowel disease (IBD). These effects are primarily mediated by autonomic neuropathy, alterations in gut hormones, gut microbial dysbiosis, and release of pro-inflammatory cytokines [48]. For example, diarrhea is a common complication of diabetes mellitus and is mainly caused by vagal nerve dysfunction and interstitial neuronal cell reduction [49]. In obese patients, a chronic low-grade inflammatory state has also been identified as one of the risk factors for the promotion of IBD, and there is a trend toward epidemiological co-morbidity between these two groups of diseases [50]. Notably, certain medications for metabolic diseases (e.g., liraglutide and semaglutide) may induce gastrointestinal dysfunction [51]. DF contributes to preventing certain metabolic disorders and bowel dysfunction [52,53].

IDF influences food intake regulation, weight management, and insulin sensitivity improvement through its physical properties. These include water absorption, increased gastrointestinal content volume, accelerated intestinal motility, and delayed gastric emptying, thereby potentially mitigating metabolic diseases [54]. SDF regulates glucose and lipid metabolism via gel formation and viscosity enhancement while functioning as a prebiotic. It serves as a fermentation substrate, promoting beneficial microbiota growth and inhibiting potentially pathogenic bacteria. It stimulates microbial production of specific metabolites—particularly BAs, SCFAs, branched-chain amino acids, trimethylamine N-oxide, tryptophan, and indole derivatives—associated with metabolic disorder pathogenesis [55,56]. SDF also modulates tight junction protein expression to reduce intestinal permeability and minimize lipopolysaccharide (LPS) translocation into circulation [57]. Additionally, it may alleviate bowel disorders, especially constipation, through the gut–microbiota–brain axis [58].

**Figure 2:**
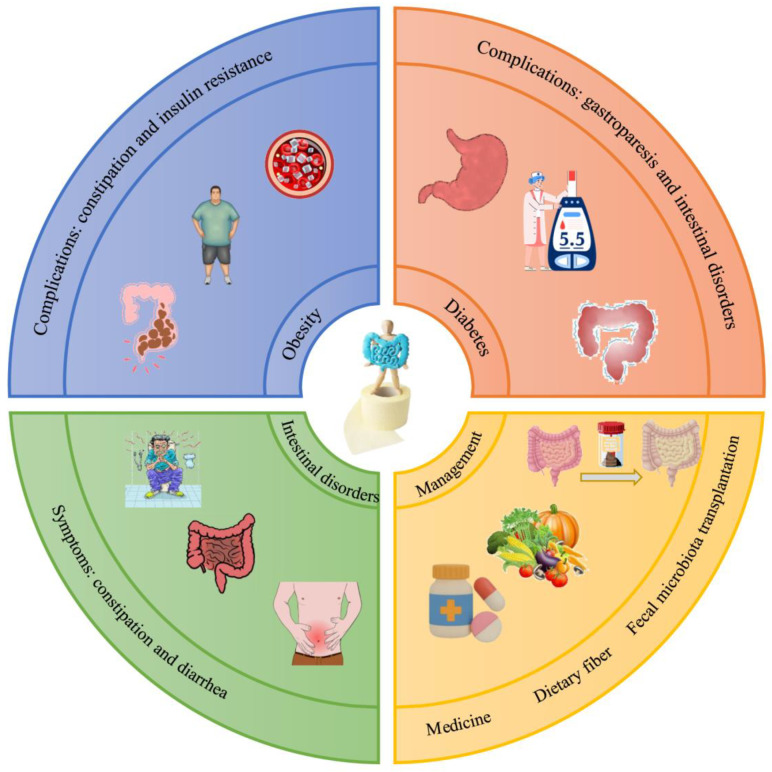
Intestinal-function-related metabolic diseases and their management.

Constipation is a common intestinal symptom that often occurs as a complication of metabolic diseases. The prevalence of moderate to severe and mild to moderate constipation in hospitalized patients with diabetes has been reported as high as 55.56% and 39.33%, respectively [59,60]. Obesity is associated with an increased risk of constipation, and the prevalence of constipation in obese adults of grades II and III is relatively high [61,62]. In addition, recent evidence suggests that constipation is independently associated with adverse clinical outcomes, such as cardiovascular disease and mortality [63]. According to health-related quality of life studies, patients with constipation are prone to severe decline in the quality of life, with the impacts being comparable to those of depression and neurological illnesses [64]. The intervention mechanism involves improving fecal morphological parameters and regulating the structure of gut bacteria and expression levels of signaling molecules. In terms of lifestyle interventions, DF is an effective means of alleviating metabolic and intestinal disorders. For severely ill patients, however, pharmacological treatment or fecal microbiota transplantation (FMT) might be a necessary option (Figure 2).

## 3. DF in the Management of Intestinal Health

According to the pathogenesis, constipation can be divided into primary and secondary constipation. Primary constipation is caused by intestinal dysfunction that makes it hard to pass feces out of the body without structural abnormalities or metabolic disorders [65]. The Rome criteria were created for the standardized definition of primary constipation. According to the latest version of the Rome IV criteria, constipation is generally classified into defecation disorder (DD), constipation-dominated irritable bowel syndrome (IBS-C), and functional constipation (FC; Table 1) [66]. Secondary constipation refers to the constipation caused by structural changes in the organ or tissue, which is often accompanied by diseases such as diabetes, adiposity, and Parkinson’s disease [67]. It is mainly caused by decreased bowel movement, which might be attributed to increased sympathetic tension and decreased parasympathetic excitability [68]. The most effective approach for secondary constipation is to address and manage the underlying conditions responsible for it, which can be done by thorough and detailed patient evaluation [69].

### 3.1. Effect of DF in Relieving Intestinal Disorder

DF relieves constipation by increasing the water content and weight of feces, which is attributed to the solubility and fermentation of DF. IDF could stimulate intestinal mucosal secretion and SDF could form gels to resist water loss [1]. Undigested DF and gut microbiota are the main causes for the increase in weight in feces [1,70]. Some trials investigating the effect of DF on constipation are shown in Table 2.

In the large intestine, DF increases fecal water content by stimulating intestinal mucosal secretion via IDF and preventing water loss by forming a gel via SDF [80]. In a clinical trial, 12 healthy volunteers who ate 37.5 g of wheat bran increased fecal humidity because of the large/coarse fiber [74]. In rats fed diets, compared with baseline, supplementation with 10% wheat bran increased fecal water content from 76.3 ± 0.91 to 80.2 ± 0.56% and hydrated fecal mass from 25.4 ± 1.5 to 49.3 ± 3.5 g per 100 g diet [71]. SDF contains a large number of hydroxyl groups, which can be physically cross-linked and form gel networks to resist colon water absorption [81]. Psyllium, a non-fermentable but gel-forming fiber, was 3.4 times more effective than wheat bran in increasing fecal production [82]. The same amount of cellulose and psyllium was offered to adult cats, and better fecal scores (including total fecal wet weight and fecal water) were observed in cats consuming psyllium [72]. Similar results were found in human experiments. In the clinical trial of 21 healthy volunteers provided with 20 g of coarse bran or fine bran, which came from the same wheat fiber but with different sizes, the fecal water content in the fine bran group was lower than that in the coarse bran group [83]. These results indicated that IDF, which are large/coarse fibers rather than fine fibers, could stimulate intestinal mucosal secretion. In a controlled trial with 29 volunteers, magnetic resonance imaging revealed significantly higher colonic water content following psyllium supplementation compared to placebo administration, suggesting that psyllium could bind more water molecules due to gel formation [76]. A study providing psyllium to both healthy participants and constipation patients observed an increase in fecal water content, and significant changes in intestinal bacteria composition and SCFA in constipation patients [75].

There are two ways that DF increases fecal weight, including undigested fiber of IDF and altered gut microbiota by SDF. IDF is the main cause of increased fecal weight and volume, as it cannot be digested and absorbed by the intestines. Consumption of kiwifruit skin with flesh significantly increases the fecal capacity-enhancing potential of the whole kiwifruit by 40–180% compared to consumption of the flesh alone [73]. In a double-blind randomized crossover trial, volunteers were provided with 10 days of a meal box containing an additional 20 g of wheat fiber or control food products, and the wet/dry feces showed an increase of 1.41 and 1.55 times, respectively, compared to the control [77]. Fecal weight increases significantly when fiber is ingested in solid form, with no change in fecal weight if it is consumed in liquid form [78]. It is possible that the structure of the fiber in the drink is disrupted to the extent that it affects the WSC. Unlike IDF, SDF primarily increases fecal weight by enhancing the richness and biomass of the gut microbiota through fermentation. Compared with healthy individuals, the species richness was reduced in patients with constipation, and some bacterial abundances were relatively reduced, such as *Lactobacilli* and *Bifidobacteria* [84]. DFs, including oligofructose, inulin, and oligogalactose, have shown prebiotic properties that refer to the colonization of beneficial gut bacteria, resulting in a significant increase in *Bifidobacterium* and/or *Lactobacillus spp.* in the gut [85]. Compared to a placebo group taking maltodextrin, a polysaccharide that can be rapidly digested and absorbed, resistant maltodextrin (RMD) increased total bacterial biomass, but the increase was dosage-dependent [79]. The gut microbiota of constipated patients was restored after DF intervention. In a meta-analysis involving 64 studies, DFs were found to elevate gut microbiota abundance, and interventions involving fructans and oligogalactans significantly increased the abundance of *Bifidobacterium* and *Lactobacillus* spp. [86].

### 3.2. Molecular Mechanism of DF in Relieving Intestinal Disorder

**Figure 3:**
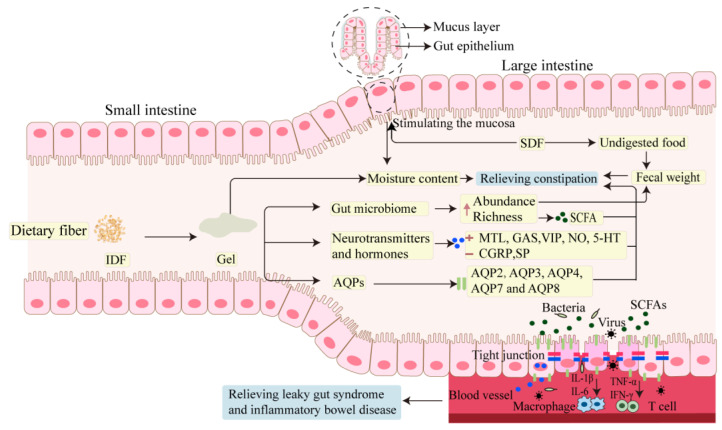
The principle of dietary fiber for the mitigation of intestinal dysfunction.

The pathogenesis of constipation is multifactorial, including colonic motility, fluid transport, and microbial alterations, as well as dietary and behavioral influences [87]. Neurotransmitters and hormones function as signaling molecules that regulate colonic motility, while aquaporins (AQPs) serve as an important channel for water transport within the intestine. Additionally, SCFAs, which are primary metabolites of gut microbiota, significantly influence intestinal motility [87]. Changes in the expression levels of these factors are closely related to constipation, which could be modified by DF. The molecular mechanisms by which DF could alter gastrointestinal neurotransmitters and hormones, AQPs, and SCFAs to relieve gastrointestinal diseases (leaky gut syndrome and IBD) are illustrated in Figure 3.

#### 3.2.1. The Effect of DF on Gastrointestinal Neurotransmitters and Hormones

Slower bowel peristalsis is one of the features of chronic constipation and prolongs the transport time [88], which is associated with abnormal secretion of gastrointestinal neurotransmitters and hormones. Enteric neurotransmitters are a class of active small-molecule peptides that transmit messages between neurons and can be divided into inhibitory and excitatory neurotransmitters. Inhibitory neurotransmitters can induce smooth muscle relaxation, while excitatory neurotransmitters stimulate contraction of intestinal muscles [88]. Hormones have important physiological significance in the regulation of gastrointestinal motility. Motilin (MTL) and gastrin (GAS) stimulate the peristalsis, while calcitonin-gene-related peptide (CGRP) and somatostatin (SS) inhibit intestinal motility [87].

Neurotransmitters and hormones differ in healthy and constipated individuals. In a study of adults with constipation, plasma levels of serum serotonin (5-HT), GAS, and vasoactive intestinal peptide (VIP) were reduced, and growth inhibitor levels increased in patients with constipation [89]. Constipated mice had lower levels of MTL, GAS, and substance P (SP) than healthy mice, and their intestinal transit rate was also lower [90]. A similar finding was found in rats, which showed significantly lower levels of MTL and SP while significantly higher amounts of CGRP and VIP in the serum in a rat model receiving drug-induced constipation [91]. These studies implied that abnormal expression of neurotransmitters and hormones may have an impact on bowel movement.

Constipation caused by neurotransmitters and hormones can be improved by DF intervention, which has been demonstrated in animal experiments [90,91,92]. When mice with loperamide-hydrochloride-induced constipation were given varying doses of hawthorn SDF (HSDF), their constipation was relieved by elevating the levels of excitatory hormones in the gastrointestinal tract (MTL, GAS, and SP) and lowering the levels of inhibitory hormones (SS, nitric oxide, and malondialdehyde), in comparison to the group without HSDF treatment [93]. The combination of sucrose and *Latilactobacillus sakei* Furu 2019 was used for constipated mice, which increased the expression of neurotrophic factors derived from glial cells associated with bowel movements, such as 5-HT and SP [94]. It was also verified in a rat constipation model. Prebiotics inulin (INU) and isomaltose oligosaccharides (IMO) significantly increased the weight and water content of feces of constipated rats [91]. The levels of gastrointestinal motility promoting factors, such as MTL and SP, were increased, while the inhibitory factors, including VIP and CGRP, were lower in INU- or IMO-fed rats than in rats without INU [91]. Providing constipated rats with a novel synbiotic, which was synthesized by GOS, stachyose, and probiotics, could significantly improve the constipation indicators (the time of the first black feces and fecal water content) and elevate the serum level of excitatory transmitters (SP, VIP, MTL, and GAS) [95]. It was found that RS and konjac flour (KON) increased bowel frequency by increasing serum 5-HT, MTL, and acetylcholine, while decreasing levels of the inhibitory neurotransmitter NO [92]. Therefore, DF can improve constipation by modulating gut motility-related factors, including gastrointestinal hormones and neurotransmitters (Figure 3).

#### 3.2.2. The Effect of DF on AQPs

Constipation is closely related to a disorder in the colonic fluid transport system. Water absorption in the gut is through paracellular and transcellular pathway transport. Transcellular transport becomes the main route of colonic fluid absorption, which involves diffusion through AQPs, passive diffusion, and cotransport [96]. AQPs play an important role in maintaining fluid homeostasis as specialized channels for the rapid transport of water molecules and small solute [87].

AQPs are a class of water channel proteins, which are associated with constipation, with 13 types of AQPs identified in mammals (AQP0–AQP12) [97]. In the constipation mouse model, the expressions of AQPs have changed, including upregulated AQP3, AQP4, and AQP8, and downregulated AQP9 [98,99]. In the rat constipation model, the expression of AQP8 was increased in the colon, while increased AQP3 expression was increased at the proximal but decreased at the distal [100]. In human, the expressions of AQP3 and AQP8 were upregulated in patients with constipation, whereas the expressions of AQP1, AQP7, and others were downregulated [96]. These studies suggest that differences in the expressions of AQPs are present in constipated patients or animals.

The expression of different types of AQPs can affect normal bowel movements, which can be regulated by DF. Partially hydrolyzed guar gum (PHGG), as a kind of SDF, is used to treat constipation in rats. It was found that 5% PHGG treatment resulted in a significant increase in the water content of rat feces and a significant decrease in the expression level of AQP3 in the colon, demonstrating that AQP3 might control the flow of water molecules through the effect of PHGG [101]. Bacterial-derived DF has a comparable impact. Rats fed with bacterial cellulose, a naturally occurring DF derived from bacteria, improved constipation symptoms, shortened defecation periods, increased feces weight, and decreased levels of inhibitory neurotransmitters and AQPs (AQP2, AQP3, and AQP4) in comparison to the constipation group [102]. More alternatives for treating constipation have emerged with the development of novel DF. Rats suffering from constipation were given varying dosages of high-specific-volume polysaccharide (HSVP), a novel type of DF that was isolated from the *Artemisia sphaerocephala Krasch* seeds. It showed that both medium- and high-dose HSVP effectively alleviated constipation, reduced the expression of intestinal AQP3, and increased the expression of the VIP-cAMP-PKA-AQP3 signaling pathway [103]. Therefore, DF has great potential to regulate the expression levels of AQPs. AQPs regulate the transmembrane transport of water molecules and play key roles in intestinal absorption, secretion, and water metabolism, thus changes in AQPs induced by DF can lead to improvement of constipation [87].

#### 3.2.3. The Effect of DF on SCFAs

SCFAs are the main metabolite of gut microbiota via DF fermentation, which influence the peristalsis and contraction of intestinal smooth muscle by reducing intestinal pH and promoting the production of intestinal peristalsis-related factors to relieve constipation [104]. The main SCFAs are acetate, propionate, and butyrate in the human intestine, with a relative ratio of 3:1:1 [105].

Changes in the proportion and concentration of SCFAs can result in constipation. In patients suffering from constipation, decreased levels of acetate and propionate were detected, and their levels were negatively correlated with the severity of constipation [106]. In another clinical trial, three SCFAs were reduced, and their ratios were significantly altered in patients with constipation [107]. A link between the content of SCFAs and constipation was also found in a rat model [108]. These results indicate that alterations in SCFAs may be one of the causes of constipation.

The concentrations of SCFAs were changed in constipated patients and in mice following DF supplementation, with constipation symptoms reversed [75,109]. When constipated mice were given soybean-residue-extracted fiber, the fecal volume and water content increased, as well as the overall content of SCFAs, which in turn stimulated intestinal motility and alleviated constipation [110]. After a one-month intervention with SCFA-acylated starch, constipated mice demonstrated significant improvement in constipation indices, and the levels of acetate and butyrate in the feces and the fecal bacteria that produce acetic and butyrate were increased, indicating that acetate and butyrate are involved in the relief of constipation [111]. High-fiber diet had a greater effect on SCFAs than gut bacteria, and the levels of 3-indole-3-pyruvate and indole-3-pyruvate were highly correlated with ET-1, SP, and MTL levels, which returned to normal levels after treatment in constipated mice [112,113]. However, there have been conflicting findings about how the concentrations of SCFAs affect intestinal motility. Acetate, butyrate, and propionate were significantly higher in fecal samples of constipated aged rats than in normal rats [108]. Fecal samples from patients with improved constipation showed reductions in acetic acid, propionic acid, and butyric acid [114]. It was found that 2.5 mM butyrate treatment inhibited the proliferation of nerve cells, affected normal cell cycles, and impaired intestinal nerve cell repair [115]. The relationship between SCFAs and constipation is variable in different studies, and this controversy may be related to factors such as the concentration, the chemical nature and dosage of SCFAs, the reactivity of the colonic segments, and the species [104]. It is important to consider individual differences when regulating constipation using SCFAs, such as the age and gender of patients.

## 4. Conclusions and Prospects

As a natural prebiotic, DF shows significant potential for relieving intestinal disorders and related metabolic diseases. In particular, DF might alleviate obesity, diabetes, and CVD by delaying glucose absorption and maintaining the gut microbial flora balance. For instance, DF could enhance the fecal water content by stimulating intestinal mucosal secretion through IDF and reducing water loss through SDF. Additionally, DF increases fecal weight by undigested fiber from IDF and by the proliferation of gut microbiota via SDF fermentation. From a molecular perspective, DF regulates the bowel movement by modulating the secretion of gastrointestinal hormones and neurotransmitters, the expression of AQPs, and the production of SCFAs.

It is recommended that the daily intake for persons aged 19–50 years is 38 g/day for men and 25 g/day for women [8]. Although this recommendation applies to most people, different individuals have different tolerance to DF and consumption of DF needs to be adjusted according to the digestive process [116,117]. Combinations of different types of DF might be a good choice. Consuming well-mixed fiber to reduce its retention time in the small intestine and prolong the retention time in the colon will lower food absorption rates, contributing to the management of the postprandial blood glucose level. Additionally, DF with prebiotic properties could be used in conjunction with probiotics to promote the colonization of probiotics with maximum efficiency, helping to prevent and control metabolic disorders. This strategy can be further enhanced using DF encapsulation technologies, such as encapsulated SDF, to better control the release rates of both fiber and probiotics. A multilayer encapsulation system can be employed, where the inner layer contains vacuum freeze-dried probiotics as the active ingredient, the middle layer consists of prebiotic matrices (inulin and β-glucan) to provide substrates for gut microbiota fermentation, and the outer layer is coated with a sodium alginate-chitosan composite to maintain structural integrity. Furthermore, different inner layer components can be tailored to target specific diseases. For instance, in the case of diabetes, *A. muciniphila* can be combined with arabinoxylan and RS3, with AXOS (arabinoxylan oligosaccharides) rapidly fermenting to promote butyrate production, which in turn stimulates L-cell-mediated intestinal barrier repair, while RS3 provides sustained energy to maintain microbiota homeostasis.

## Figures and Tables

**Table 1:** ROME-IV diagnostic criteria for intestinal disorder.

Diagnostic Criteria for FC	Diagnostic Criteria for IBS-C	Diagnostic Criteria for DD
1. Must include ≥2 of the following:	1. Recurrent abdominal pain at least 1 day/week with ≥2 of the following:	1. The patient satisfies diagnostic criteria for FC and/or IBS-C.
a. >25% of defecations will be strained.		
b. Lumpy or hard feces > 25% of defecations.	a. Related to defecation.	2. During repeated attempts to defecate, the patient must have ≥2 of the following:
c. >25% of defecations feel like incomplete evacuation.	b. Related to change in frequency of stools.	
d. >25% of defecations feel anorectalobstruction/obstruclion.	c. Related to change in form of stools.	a. Abnormal balloon expulsion test.
e. Manual maneuvers to facilitate >25% of defecations.	2. Lumpy or hard stools > 25% of defecations.	b. Abnormal anorectal evacuation pattern with manometry or anal surface electromiography.
f. Spontaneous defecations < 3/week.		
2. Loose stools are rarely present without the use of laxatives.		c. Impaired rectal evacuation by imaging.
3. Insufficient criteria for IBS.		

Abbreviations: FC, functional constipation; IBS-C, constipation-dominated irritable bowel syndrome; DD, defecation disorder.

**Table 2:** Summary of the trials investigating the effect of DF on intestinal disorder.

Subjects	Number	Study Design	Intervention	Comparator	Duration	Outcomes	References
Adult rats	*n* = 8, males	Crossover	Diet with 10% wheat bran, adding different doses of psyllium/psyllium/guar gum/raftilose	Diet with 10% wheat bran	7 days	Increased fecal hydration capacity, increasing by 2.4 ± 0.29 g per gram of wheat bran ingested, and by 15.6 ± 1.52 g per g of psyllium	[71]
Healthy adult cats	Female (*n* = 6) and male (*n* = 3)	RCT	Dry extruded diet containing 6% psyllium	Dry extruded diet containing 6% cellulose	10 days	The mean fecal score was higher (*p* < 0.0001) for the control vs. intervention group; the total fecal wet weight (*p* = 0.0003) and fecal moisture (%) were also higher (*p* = 0.0426) for the intervention group	[72]
Rats	*n* = 8	Crossover RCT	Daily diet, adding skin or flesh of four kiwifruit cultivars/wheat bran	Daily diet	7 days	Increasing the abundance of *Lachnospiraceae* and *Lactobacillus* spp. and three kiwifruit cultivars increased the fecal dry weight (*p* < 0.001)	[73]
Healthy adults	*n* = 10, males	Crossover	Normal diet + 37.5 g wheat bran	Normal diet	10 days	Improved fecal weight (*p* < 0.05) and reduced gut transit time in intervention compared to normal diet (*p* < 0.05)	[74]
Healthy adults and constipatied patients	Healthy adults (*n* = 8), adults with chronic constipation (*n* = 16)	RCT	Diet with psyllium	Diet with maltodextrin	7 days	Increased fecal water content in the control group of constipated patients and increased *Lachnospira*, *Roseburia*, and *Faecalibacterium* in healthy adults, with *Veillonella* and *Subdoligranulum* showing changes	[75]
Healthy adults and constipatied patients	Healthy adults (*n* = 9), constipated patients (*n* = 24)	Crossover RCT	Patients took maltodextrin (placebo) and psyllium 7 g	Controls group took three treatments in randomized order—placebo, psyllium 3.5 g, and 7 g	6 days	Increased fasting colonic volumes (*p* < 0.05) and mean postprandial small bowel water in control and intervention groups after taking 7 g of psyllium	[76]
Healthy adults	*n* = 16, males	Crossover RCT	Normal diet with different doses of extrinsic wheat fiber	Normal diet	10 days	Increased feces wet and dry weight compared to control (*p* < 0.01) and increased stool frequency from 1.1 ± 0.1 defecations per day to 1.3 ± 0.1 defecations per day (*p* < 0.05)	[77]
Healthy adults	Female (*n* = 5) and male (*n* = 5)	Crossover RCT	Normal diet with 10 g wheat fiber	Normal diet	5 days	Increased fecal wet weight (*p* < 0.05)	[78]
Healthy adults	*n* = 14, males	Crossover RCT	25 g/d RM + 25 g/d placebo and 50 g/d RM + 0 g/d placebo	50 g/d placebo	24 days	Increased fecal wet weight (*p* < 0.0001) and fecal dry weight (*p* < 0.0001) compared with the placebo group, and total counts of fecal bacteria increased by 12% (*p* = 0.17) and 18% (*p* = 0.019), respectively	[79]

Abbreviations: RCT, randomized controlled trial; RM, resistant maltodextrin.

## Data Availability

No new data were created or analyzed in this study.

## References

[1] Gill S.K., Rossi M., Bajka B., Whelan K. (2020). Dietary fibre in gastrointestinal health and disease. Nat. Rev. Gastroenterol. Hepatol..

[2] Tang W., Lin X.Y., Walayat N., Liu J., Zhao P. (2023). Dietary fiber modification: Structure, physicochemical properties, bioactivities, and application—A review. Crit. Rev. Food Sci..

[3] Kumar R., Butreddy A., Kommineni N., Reddy P.G., Bunekar N., Sarkar C., Dutt S., Mishra V.K., Aadil K.R., Mishra Y.K. (2021). Lignin: Drug/Gene Delivery and Tissue Engineering Applications. Int. J. Nanomed..

[4] Ye S.X., Shah B.R., Li J., Liang H.S., Zhan F.C., Geng F., Li B. (2022). A critical review on interplay between dietary fibers and gut microbiota. Trends Food Sci. Technol..

[5] Guan Z.W., Yu E.Z., Feng Q. (2021). Soluble Dietary Fiber, One of the Most Important Nutrients for the Gut Microbiota. Molecules.

[6] Han X.B., Ma Y., Ding S.J., Fang J., Liu G. (2023). Regulation of dietary fiber on intestinal microorganisms and its effects on animal health. Anim. Nutr..

[7] Xue Y., Cui L., Qi J., Ojo O., Du X., Liu Y., Wang X. (2021). The effect of dietary fiber (oat bran) supplement on blood pressure in patients with essential hypertension: A randomized controlled trial. Nutr. Metab. Cardiovasc. Dis..

[8] Bakr A.F., Farag M.A. (2023). Soluble Dietary Fibers as Antihyperlipidemic Agents: A Comprehensive Review to Maximize Their Health Benefits. ACS Omega.

[9] Delzenne N.M., Olivares M., Neyrinck A.M., Beaumont M., Kjolbaek L., Larsen T.M., Benitez-Paez A., Romani-Perez M., Garcia-Campayo V., Bosscher D. (2020). Nutritional interest of dietary fiber and prebiotics in obesity: Lessons from the MyNewGut consortium. Clin. Nutr..

[10] Oniszczuk A., Oniszczuk T., Gancarz M., Szymanska J. (2021). Role of Gut Microbiota, Probiotics and Prebiotics in the Cardiovascular Diseases. Molecules.

[11] Nie Y., Luo F.J. (2021). Dietary Fiber: An Opportunity for a Global Control of Hyperlipidemia. Oxid. Med. Cell Longev..

[12] Beccard S., Bernard J., Wouters R., Gehrich K., Zielbauer B., Mezger M., Vilgis T.A. (2019). Alteration of the structural properties of inulin gels. Food Hydrocolloid..

[13] Kadyan S., Sharma A., Arjmandi B.H., Singh P., Nagpal R. (2022). Prebiotic Potential of Dietary Beans and Pulses and Their Resistant Starch for Aging-Associated Gut and Metabolic Health. Nutrients.

[14] Brownlee I.A., Allen A., Pearson J.P., Dettmar P.W., Havler M.E., Atherton M.R., Onsoyen E. (2005). Alginate as a source of dietary fiber. Crit. Rev. Food Sci. Nutr..

[15] Dong H., Sun Y., Nie L., Cui A., Zhao P., Leung W.K., Wang Q. (2024). Metabolic memory: Mechanisms and diseases. Signal Transduct. Target. Ther..

[16] Ioniță-Mîndrican C.-B., Ziani K., Mititelu M., Oprea E., Neacșu S.M., Moroșan E., Dumitrescu D.-E., Roșca A.C., Drăgănescu D., Negrei C. (2022). Therapeutic Benefits and Dietary Restrictions of Fiber Intake: A State of the Art Review. Nutrients.

[17] Cicero A.F.G., Fogacci F., Bove M., Giovannini M., Borghi C. (2021). Impact of a short-term synbiotic supplementation on metabolic syndrome and systemic inflammation in elderly patients: A randomized placebo-controlled clinical trial. Eur. J. Nutr..

[18] Singh R., Zogg H., Wei L., Bartlett A., Ghoshal U.C., Rajender S., Ro S. (2021). Gut Microbial Dysbiosis in the Pathogenesis of Gastrointestinal Dysmotility and Metabolic Disorders. J. Neurogastroenterol. Motil..

[19] Wei L., Ji L., Miao Y., Han X., Li Y., Wang Z., Fu J., Guo L., Su Y., Zhang Y. (2023). Constipation in DM are associated with both poor glycemic control and diabetic complications: Current status and future directions. Biomed. Pharmacother..

[20] Al-Habsi N., Al-Khalili M., Haque S.A., Elias M., Olqi N.A., Al Uraimi T. (2024). Health Benefits of Prebiotics, Probiotics, Synbiotics, and Postbiotics. Nutrients.

[21] Li H.Y., Zhou D.D., Gan R.Y., Huang S.Y., Zhao C.N., Shang A., Xu X.Y., Li H.B. (2021). Effects and Mechanisms of Probiotics, Prebiotics, Synbiotics, and Postbiotics on Metabolic Diseases Targeting Gut Microbiota: A Narrative Review. Nutrients.

[22] Veluvali A., Snyder M. (2023). Dietary fiber deficiency in individuals with metabolic syndrome: A review. Curr. Opin. Clin. Nutr..

[23] Liu J., Yu L.L., Wu Y. (2020). Bioactive Components and Health Beneficial Properties of Whole Wheat Foods. J. Agric. Food. Chem..

[24] Gona P.N., Kimokoti R.W., Gona C.M., Ballout S., Rao S.R., Mapoma C.C., Lo J., Mokdad A.H. (2021). Changes in body mass index, obesity, and overweight in Southern Africa development countries, 1990 to 2019: Findings from the Global Burden of Disease, Injuries, and Risk Factors Study. Obes. Sci. Pr..

[25] Piche M.E., Tchernof A., Despres J.P. (2020). Obesity Phenotypes, Diabetes, and Cardiovascular Diseases. Circ. Res..

[26] Vallianou N., Stratigou T., Christodoulatos G.S., Tsigalou C., Dalamaga M. (2020). Probiotics, Prebiotics, Synbiotics, Postbiotics, and Obesity: Current Evidence, Controversies, and Perspectives. Curr. Obes. Rep..

[27] Huwiler V.V., Schönenberger K.A., von Brunegg A.S., Reber E., Mühlebach S., Stanga Z., Balmer M.L. (2022). Prolonged Isolated Soluble Dietary Fibre Supplementation in Overweight and Obese Patients: A Systematic Review with Meta-Analysis of Randomised Controlled Trials. Nutrients.

[28] Li H.T., Zhang L., Li J., Wu Q., Qian L.L., He J.S., Ni Y.Q., Kovatcheva-Datchary P., Yuan R., Liu S.B. (2024). Resistant starch intake facilitates weight loss in humans by reshaping the gut microbiota. Nat. Metab..

[29] Jovanovski E., Mazhar N., Komishon A., Khayyat R., Li D., Blanco Mejia S., Khan T., L Jenkins A., Smircic-Duvnjak L., Sievenpiper J.L. (2020). Can dietary viscous fiber affect body weight independently of an energy-restrictive diet? A systematic review and meta-analysis of randomized controlled trials. Am. J. Clin. Nutr..

[30] Deehan E.C., Zhang Z.X., Riva A., Armet A.M., Perez-Muñoz M.E., Nguyen N.K., Krysa J.A., Seethaler B., Zhao Y.Y., Cole J. (2022). Elucidating the role of the gut microbiota in the physiological effects of dietary fiber. Microbiome.

[31] Freire R.H., Alvarez-Leite J.I. (2020). Appetite control: Hormones or diet strategies?. Curr. Opin. Clin. Nutr. Metab. Care.

[32] Akhlaghi M. (2024). The role of dietary fibers in regulating appetite, an overview of mechanisms and weight consequences. Crit. Rev. Food Sci. Nutr..

[33] Sun H., Saeedi P., Karuranga S., Pinkepank M., Ogurtsova K., Duncan B.B., Stein C., Basit A., Chan J.C.N., Mbanya J.C. (2023). IDF Diabetes Atlas: Global, regional and country-level diabetes prevalence estimates for 2021 and projections for 2045. Diabetes Res. Clin. Pr..

[34] Wu S.M., Jia W., He H.M., Yin J., Xu H.L., He C.Y., Zhang Q.Q., Peng Y., Cheng R.Y. (2023). A New Dietary Fiber Can Enhance Satiety and Reduce Postprandial Blood Glucose in Healthy Adults: A Randomized Cross-Over Trial. Nutrients.

[35] Kokubo E., Morita S., Nagashima H., Oshio K., Iwamoto H., Miyaji K. (2022). Blood Glucose Response of a Low-Carbohydrate Oral Nutritional Supplement with Isomaltulose and Soluble Dietary Fiber in Individuals with Prediabetes: A Randomized, Single-Blind Crossover Trial. Nutrients.

[36] Chen L.H., Liu B., Ren L.X., Du H., Fei C.H., Qian C., Li B., Zhang R.X., Liu H.X., Li Z.J. (2023). High-fiber diet ameliorates gut microbiota, serum metabolism and emotional mood in type 2 diabetes patients. Front. Cell Infect. Mi..

[37] Zhao L.P., Zhang F., Ding X.Y., Wu G.J., Lam Y.Y., Wang X.J., Fu H.Q., Xue X.H., Lu C.H., Ma J.L. (2018). Gut bacteria selectively promoted by dietary fibers alleviate type 2 diabetes. Science.

[38] He Y., Wang B., Wen L., Wang F., Yu H., Chen D., Su X., Zhang C. (2022). Effects of dietary fiber on human health. Food Sci. Hum. Wellness.

[39] Wu H.C., Chiou J.C. (2021). Potential Benefits of Probiotics and Prebiotics for Coronary Heart Disease and Stroke. Nutrients.

[40] Ribas S.A., Cunha D.B., Sichieri R., da Silva L.C.S. (2015). Effects of psyllium on LDL-cholesterol concentrations in Brazilian children and adolescents: A randomised, placebo-controlled, parallel clinical trial. Br. J. Nutr..

[41] González A.P., Flores-Ramírez A., Gutiérrez-Castro K.P., Luévano-Contreras C., Gómez-Ojeda A., Sosa-Bustamante G.P., Caccavello R., Barrera-de León J.C., Garay-Sevilla M.E., Gugliucci A. (2021). Reduction of small dense LDL and Il-6 after intervention with Plantago psyllium in adolescents with obesity: A parallel, double blind, randomized clinical trial. Eur. J. Pediatr..

[42] Liu L., Shi Z., Ji X., Zhang W., Luan J., Zahr T., Qiang L. (2022). Adipokines, adiposity, and atherosclerosis. Cell Mol. Life Sci..

[43] Zou X., Xu X., Chao Z., Jiang X., Zheng L., Jiang B. (2022). Properties of plant-derived soluble dietary fibers for fiber-enriched foods: A comparative evaluation. Int. J. Biol. Macromol..

[44] Ranaivo H., Thirion F., Bèra-Maillet C., Guilly S., Simon C., Sothier M., Van den Berghe L., Feugier-Favier N., Lambert-Porcheron S., Dussouse I. (2022). Increasing the diversity of dietary fibers in a daily-consumed bread modifies gut microbiota and metabolic profile in subjects at cardiometabolic risk. Gut Microbes.

[45] Sabbione A.C., Anon M.C., Scilingo A. (2024). Characterization and Bile Acid Binding Capacity of Dietary Fiber Obtained from Three Different Amaranth Products. Plant Foods Hum. Nutr..

[46] de Vos W.M., Tilg H., Van Hul M., Cani P.D. (2022). Gut microbiome and health: Mechanistic insights. Gut.

[47] Viuda-Martos M., López-Marcos M.C., Fernández-López J., Sendra E., López-Vargas J.H., Pérez-Alvarez J.A. (2010). Role of Fiber in Cardiovascular Diseases: A Review. Compr. Rev. Food. Sci. F.

[48] Mare R., Sporea I. (2022). Gastrointestinal and Liver Complications in Patients with Diabetes Mellitus-A Review of the Literature. J. Clin. Med..

[49] Camilleri M. (2021). Gastrointestinal motility disorders in neurologic disease. J. Clin. Investig..

[50] Singh S., Dulai P.S., Zarrinpar A., Ramamoorthy S., Sandborn W.J. (2017). Obesity in IBD: Epidemiology, pathogenesis, disease course and treatment outcomes. Nat. Rev. Gastroenterol. Hepatol..

[51] Gudzune K.A., Kushner R.F. (2024). Medications for Obesity: A Review. JAMA.

[52] Megur A., Daliri E.B., Baltriukiene D., Burokas A. (2022). Prebiotics as a Tool for the Prevention and Treatment of Obesity and Diabetes: Classification and Ability to Modulate the Gut Microbiota. Int. J. Mol. Sci..

[53] Gong L., Cao W., Chi H., Wang J., Zhang H., Liu J., Sun B. (2018). Whole cereal grains and potential health effects: Involvement of the gut microbiota. Food Res. Int..

[54] Salvatore S., Battigaglia M.S., Murone E., Dozio E., Pensabene L., Agosti M. (2023). Dietary Fibers in Healthy Children and in Pediatric Gastrointestinal Disorders: A Practical Guide. Nutrients.

[55] Canfora E.E., Jocken J.W., Blaak E.E. (2015). Short-chain fatty acids in control of body weight and insulin sensitivity. Nat. Rev. Endocrinol..

[56] Gong L., Liu F., Liu J., Wang J. (2024). Dietary fiber (oligosaccharide and non-starch polysaccharide) in preventing and treating functional gastrointestinal disorders—Challenges and controversies: A review. Int. J. Biol. Macromol..

[57] Delzenne N.M., Cani P.D. (2011). Interaction between obesity and the gut microbiota: Relevance in nutrition. Annu. Rev. Nutr..

[58] Erhardt R., Harnett J.E., Steels E., Steadman K.J. (2023). Functional constipation and the effect of prebiotics on the gut microbiota: A review. Br. J. Nutr..

[59] Sangnes D.A., Lundervold K., Bekkelund M., von Volkmann H.L., Berentsen B., Gilja O.H., Dimcevski G., Softeland E. (2021). Gastrointestinal transit and contractility in diabetic constipation: A wireless motility capsule study on diabetes patients and healthy controls. United Eur. Gastroenterol. J..

[60] Ito H., Ito K., Tanaka M., Hokamura M., Tanaka M., Kusano E., Kondo J., Izutsu T., Matsumoto S., Inoue H. (2022). Constipation Is a Frequent Problem Associated with Vascular Complications in Patients with Type 2 Diabetes: A Cross-sectional Study. Intern. Med..

[61] Silveira E.A., Santos A.S.e.A.d.C., Ribeiro J.N., Noll M., dos Santos Rodrigues A.P., de Oliveira C. (2021). Prevalence of constipation in adults with obesity class II and III and associated factors. BMC Gastroenterol..

[62] Sun X., Zhang S., Zhou X. (2024). A causal association between obesity and constipation: A two-sample bidirectional Mendelian randomization study and meta-analysis. Front. Nutr..

[63] Sumida K., Yamagata K., Kovesdy C.P. (2020). Constipation in CKD. Kidney Int. Rep..

[64] Erdur B., Ayar M. (2020). The treatment of functional constipation significantly increased quality of life in children aged 4-17 years. Turk. J. Gastroenterol..

[65] Bharucha A.E., Lacy B.E. (2020). Mechanisms, Evaluation, and Management of Chronic Constipation. Gastroenterology.

[66] Caetano A.C., Costa D., Silva-Mendes S., Correia-Pinto J., Rolanda C. (2022). Constipation: Prevalence in the Portuguese community using Rome IV-Associated factors, toilet behaviours and healthcare seeking. United Eur. Gastroent..

[67] Milosavljevic T., Popovic D.D., Mijac D.D., Milovanovic T., Krstic S., Krstic M.N. (2022). Chronic Constipation: Gastroenterohepatologist’s Approach. Dig. Dis..

[68] Arco S., Saldaña E., Serra-Prat M., Palomera E., Ribas Y., Font S., Clavé P., Mundet L. (2022). Functional Constipation in Older Adults: Prevalence, Clinical Symptoms and Subtypes, Association with Frailty, and Impact on Quality of Life. Gerontology.

[69] Sharma A., Rao S., Greenwood-Van Meerveld B. (2017). Constipation: Pathophysiology and Current Therapeutic Approaches. Gastrointestinal Pharmacology.

[70] A M Stephen J.H.C. (1980). The microbial contribution to human faecal mass. J. Med. Microbiol..

[71] Monro J.A. (2024). Quantitative management of human faecal bulking response to combinations of functionally distinct dietary fibers, using functional equivalents and a validated rat model. Int. J. Food Sci. Nutr..

[72] Keller E., Laxalde J., Tranier N., von Kretschmann P.B., Jackson A., van Hoek I. (2024). Psyllium husk powder increases defecation frequency and faecal score, bulk and moisture in healthy cats. J. Feline Med. Surg..

[73] Monro J.A., Paturi G. (2020). Kiwifruit Skin and Flesh Contributions to Fecal Bulking and Bacterial Abundance in Rats. Plant Food Hum. Nutr..

[74] Tomlin J.R.N. (1988). Laxative properties of indigestible plastic particles. Br. Med. J..

[75] Jalanka J., Major G., Murray K., Singh G., Nowak A., Kurtz C., Silos-Santiago I., Johnston J.M., de Vos W.M., Spiller R. (2019). The Effect of Psyllium Husk on Intestinal Microbiota in Constipated Patients and Healthy Controls. Int. J. Mol. Sci..

[76] Major G., Murray K., Singh G., Nowak A., Hoad C.L., Marciani L., Silos-Santiago A., Kurtz C.B., Johnston J.M., Gowland P. (2018). Demonstration of differences in colonic volumes, transit, chyme consistency, and response to psyllium between healthy and constipated subjects using magnetic resonance imaging. Neurogastroenterol. Motil..

[77] de Wit N., Esser D., Siebelink E., Fischer A., Sieg J., Mes J. (2019). Extrinsic wheat fibre consumption enhances faecal bulk and stool frequency; a randomized controlled trial. Food Funct..

[78] Brandl B., Lee Y.M., Dunkel A., Hofmann T., Hauner H., Skurk T. (2020). Effects of Extrinsic Wheat Fiber Supplementation on Fecal Weight; A Randomized Controlled Trial. Nutrients.

[79] Baer D.J., Stote K.S., Henderson T., Paul D.R., Okuma K., Tagami H., Kanahori S., Gordon D.T., Rumpler W.V., Ukhanova M. (2014). The Metabolizable Energy of Dietary Resistant Maltodextrin Is Variable and Alters Fecal Microbiota Composition in Adult Men. J. Nutr..

[80] McRorie J.W., McKeown N.M. (2017). Understanding the Physics of Functional Fibers in the Gastrointestinal Tract: An Evidence-Based Approach to Resolving Enduring Misconceptions about Insoluble and Soluble Fiber. J. Acad. Nutr. Diet.

[81] Peleg-Evron O., Davidovich-Pinhas M., Bianco-Peled H. (2023). Crosslinking konjac-glucomannan with kappa-carrageenan nanogels: A step toward the design of sacrificial materials. Int. J. Biol. Macromol..

[82] McRorie J.W., Fahey G.C., Gibb R.D., Chey W.D. (2020). Laxative effects of wheat bran and psyllium: Resolving enduring misconceptions about fiber in treatment guidelines for chronic idiopathic constipation. J. Am. Assoc. Nurse Pra..

[83] Brodribb A.J.M., Groves C. (1978). Effect of Bran Particle-Size on Stool Weight. Gut.

[84] Ohkusa T., Koido S., Nishikawa Y., Sato N. (2019). Gut Microbiota and Chronic Constipation: A Review and Update. Front. Med.-Lausanne.

[85] Hughes R.L., Alvarado D.A., Swanson K.S., Holscher H.D. (2022). The Prebiotic Potential of Inulin-Type Fructans: A Systematic Review. Adv. Nutr..

[86] So D., Whelan K., Rossi M., Morrison M., Holtmann G., Kelly J.T., Shanahan E.R., Staudacher H.M., Campbell K.L. (2018). Dietary fiber intervention on gut microbiota composition in healthy adults: A systematic review and meta-analysis. Am. J. Clin. Nutr..

[87] Zhao Q., Chen Y.Y., Xu D.Q., Yue S.J., Fu R.J., Yang J., Xing L.M., Tang Y.P. (2021). Action Mode of Gut Motility, Fluid and Electrolyte Transport in Chronic Constipation. Front. Pharmacol..

[88] Wang H.L. (2015). Understanding the Pathogenesis of Slow-Transit Constipation: One Step Forward. Dig. Dis. Sci..

[89] Ge Z.Y., Duan Z.J., Yang H., Zhang S.G., Zhang S., Wang L.X., Yang D., Sun X.Y., Zhang Z.F., Su L.P. (2018). Home-Based Transcutaneous Neuromodulation Improved Constipation via Modulating Gastrointestinal Hormones and Bile Acids. Evid.-Based Complement. Altern. Med..

[90] Huang H., Wang Y.T., Ding X.F., Li F., Gu J., Zeng F.M., Jiang J., Ji L.J. (2024). Hemp seeds attenuate loperamide-induced constipation in mice. Front. Microbiol..

[91] Lan J.H., Wang K.L., Chen G.Y., Cao G.T., Yang C.M. (2020). Effects of inulin and isomalto-oligosaccharide on diphenoxylate-induced constipation, gastrointestinal motility-related hormones, short-chain fatty acids, and the intestinal flora in rats. Food Funct..

[92] Lu D.D., Pi Y., Ye H., Wu Y.J., Bai Y., Lian S., Han D.D., Ni D.J., Zou X.H., Zhao J.B. (2022). Consumption of Dietary Fiber with Different Physicochemical Properties during Late Pregnancy Alters the Gut Microbiota and Relieves Constipation in Sow Model. Nutrients.

[93] Zhang H.H., Zu Q.X., Zhang J.C., Liu S.W., Zhang G.H., Chang X.D., Li X.J. (2024). Soluble Dietary Fiber of Hawthorn Relieves Constipation Induced by Loperamide Hydrochloride by Improving Intestinal Flora and Inflammation, Thereby Regulating the Aquaporin Ion Pathway in Mice. Foods.

[94] Guo Y.A., Song L.Q., Huang Y.M., Li X.P., Xiao Y.C., Wang Z.H., Ren Z.H. (2023). Furu2019 and stachyose as probiotics, prebiotics, and synbiotics alleviate constipation in mice. Front Nutr..

[95] Yang Z.D., Ye S.M., Xu Z.M., Su H.H., Tian X., Han B., Shen B.C., Liao Q.F., Xie Z.Y., Hong Y.J. (2021). Dietary synbiotic ameliorates constipation through the modulation of gut microbiota and its metabolic function. Food Res. Int..

[96] Lin C.H., He H.Q., Kim J.J., Zheng X., Huang Z.H., Dai N. (2023). Osmotic pressure induces translocation of aquaporin-8 by P38 and JNK MAPK signaling pathways in patients with functional constipation. Dig. Liver Dis..

[97] Cai T., Dong Y., Feng Z.Y., Cai B. (2024). Ameliorative effects of the mixed aqueous extract of Aurantii Fructus Immaturus and Magnoliae Officinalis Cortex on loperamide-induced STC mice. Heliyon.

[98] Yi R.K., Peng P., Zhang J., Du M.Y., Lan L.X., Qian Y., Zhou J., Zhao X. (2019). CQPC02-Fermented Soybean Milk Improves Loperamide-Induced Constipation in Mice. J. Med. Food.

[99] Wang Y.J., Jiang H., Wang L.J., Gan H.P., Xiao X.C., Huang L.W., Li W.X., Li Z.R. (2023). Luteolin ameliorates loperamide-induced functional constipation in mice. Braz. J. Med. Biol. Res..

[100] Xi H., Youguang X., Kai H., Weiwei Q., Qing L.H., Qing Z., Jingbo X. (2023). Hetong decoction relieves loperamide-induced constipation in rats by regulating expression of aquaporins. J. Tradit. Chin. Med..

[101] Kon R., Ikarashi N., Onuma K., Yasukawa Z., Ozeki M., Sakai H., Kamei J. (2023). Effect of partially hydrolyzed guar gum on the expression of aquaporin-3 in the colon. Food Sci. Nutr..

[102] Zhai X.C., Lin D.H., Zhao Y., Yang X.B. (2018). Bacterial Cellulose Relieves Diphenoxylate-Induced Constipation in Rats. J. Agric. Food Chem..

[103] Cong L., Duan L.W., Su W.P., Hao S.H., Li D.F. (2019). Efficacy of High Specific Volume Polysaccharide—A New Type of Dietary Fiber—On Molecular Mechanism of Intestinal Water Metabolism in Rats With Constipation. Med. Sci. Monit..

[104] Wang J.K., Yao S.K. (2021). Roles of Gut Microbiota and Metabolites in Pathogenesis of Functional Constipation. Evid.-Based Complement. Altern. Med..

[105] Deehan E.C., Mocanu V., Madsen K.L. (2024). Effects of dietary fibre on metabolic health and obesity. Nat. Rev. Gastroenterol. Hepatol..

[106] Chen Q., Chen D., Gao X.Y., Jiang Y., Yu T., Jiang L.Q., Tang Y.R. (2024). Association between fecal short-chain fatty acid levels and constipation severity in subjects with slow transit constipation. Eur. J. Gastroen. Hepat..

[107] Fredericks E., Theunissen R., Roux S. (2020). Short chain fatty acids and monocarboxylate transporters in irritable bowel syndrome. Turk. J. Gastroenterol..

[108] Liu X.J., Li M.Y., Jian C., Wei F.X., Liu H.L., Li K., Qin X.M. (2022). Polysaccharide Alleviates Constipation in the Elderly Via Modification of Gut Microbiota and Fecal Metabolism. Rejuv. Res..

[109] Song H., Guo R., Sun X.B., Kou Y.X., Ma X., Chen Y.A., Song L.H., Yuan C.M., Wu Y. (2023). Xylooligosaccharides from corn cobs alleviate loperamide-induced constipation in mice modulation of gut microbiota and SCFA metabolism. Food Funct..

[110] Wu L., Tang C.H., Chen L.L., Zhao J.Y. (2023). Modified dietary fiber from soybean dregs by fermentation alleviated constipation in mice. Food Chem.-X.

[111] Wang L.L., Cen S., Wang G., Lee Y.K., Zhao J.X., Zhan H., Chen W. (2020). Acetic acid and butyric acid released in large intestine play different roles in the alleviation of constipation. J. Funct. Foods.

[112] Sinha A.K., Laursen M.F., Brinck J.E., Rybtke M.L., Hjorne A.P., Procházková N., Pedersen M., Roager H.M., Licht T.R. (2024). Dietary fibre directs microbial tryptophan metabolism via metabolic interactions in the gut microbiota. Nat. Microbiol..

[113] Duan T.C., Wang X.Y., Dong X.Y., Wang C.N., Wang L., Yang X.B., Li T. (2023). Broccoli-Derived Exosome-like Nanoparticles Alleviate Loperamide-Induced Constipation, in Correlation with Regulation on Gut Microbiota and Tryptophan Metabolism. J. Agric. Food Chem..

[114] Li X.R., Liu C.J., Tang X.D., Zhang H.M., Luo Y.Y., Zhang L., Yang E. (2020). Gut Microbiota Alterations from Three-Strain Yogurt Formulation Treatments in Slow-Transit Constipation. Can. J. Infect. Dis. Med..

[115] Wang L., Lv W.Q., Yang J.T., Lin X., Liu H.M., Tan H.J., Quan R.P., Long P.P., Shen H., Shen J. (2023). Enteric nervous system damage caused by abnormal intestinal butyrate metabolism may lead to functional constipation. Front. Microbiol..

[116] Peng A.W., Juraschek S.P., Appel L.J., Miller E.R., Mueller N.T. (2019). Effects of the DASH Diet and Sodium Intake on Bloating: Results From the DASH-Sodium Trial. Am. J. Gastroenterol..

[117] Ho K.S., Tan C.Y., Mohd Daud M.A., Seow-Choen F. (2012). Stopping or reducing dietary fiber intake reduces constipation and its associated symptoms. World J. Gastroenterol..

